# Cefepime-Induced Encephalopathy With Seizures in a Pediatric Patient With End-Stage Renal Disease Rapidly Reversed by High-Efficiency Hemodialysis

**DOI:** 10.7759/cureus.13842

**Published:** 2021-03-12

**Authors:** Siddharth Shah, Stephanie Bland

**Affiliations:** 1 Pediatric Nephrology, Norton Children's Hospital and University of Louisville, Louisville, USA; 2 Pediatrics, Norton Children's Hospital and University of Louisville, Louisville, USA

**Keywords:** cefepime, encephalopathy, high-efficiency, hemodialysis

## Abstract

Cefepime-induced encephalopathy with associated complications is a relatively rare but known adverse reaction that appears to occur more commonly in the elderly population with concomitant renal dysfunction or pre-existing central nervous system disease. The description of neurological features in pediatric patients secondary to cefepime-induced encephalopathy has rarely been reported, often delaying diagnosis and treatment. We report a 13-year-old female with end-stage renal disease, maintained on hemodialysis, who developed acute neurological symptoms of aphasia, myoclonus, hallucinations, seizures, and altered mental status after two days of cefepime treatment. After prompt discontinuation of cefepime and urgent hemodialysis, the neurological symptoms were resolved. Our patient had complete neurological recovery within 24 hours of the initial presentation of neurological symptoms. Recognizing the possibility of encephalopathy related to cefepime administration in high-risk pediatric patients should prompt close neurological monitoring when under treatment. Hemodialysis may help rapidly reverse the symptoms of cefepime-induced encephalopathy.

## Introduction

Cefepime, a commonly used fourth-generation cephalosporin, is an excellent antimicrobial for the broad-spectrum treatment and empiric coverage of gram-positive and gram-negative infectious organisms and generally reported safe in pediatric patients [1]. Cefepime is known to have neurotoxic side effects, including encephalopathy, aphasia, myoclonus, seizures, and non-convulsive status epilepticus [2]. Higher risk for neurotoxic effects has been shown in patients with older age, impaired renal function, critical illness, or pre-existing central nervous system disease [2-4]. While the treatment of cefepime-induced encephalopathy is mainly symptomatic along with discontinuation of cefepime and administration of anti-epileptic drugs (AEDs) for seizures, hemodialysis or continuous renal replacement therapy has also been reported to accelerate neurological recovery [2,5,6]. Most cases of cefepime-induced encephalopathy are reported in adults. In the literature review, we identified only four reports of cefepime-induced encephalopathy in pediatric patients, all associated with renal impairment (end-stage renal disease on dialysis or acute kidney injury [7-10]. The use of high-efficiency hemodialysis to treat cefepime encephalopathy in pediatric patients is not previously reported to our knowledge.

## Case presentation

A 13-year-old female (weight: 68 kg) with a history of major depressive and behavioral disorder, focal segmental glomerulosclerosis, and subsequent hemodialysis dependent end-stage renal disease presented after she intentionally manipulated and contaminated her hemodialysis catheter site. She was asymptomatic at the time of the presentation. The past medical history was negative for CNS abnormalities or seizures, but she had a history of central line infections. Given the high-risk of developing a blood-stream bacterial infection, screening labs and blood cultures were obtained, and empiric intravenous antibiotic therapy with cefepime was initiated. The screening labs were notable for a blood urea nitrogen (BUN) of 22 mg/dL, serum creatinine of 6.8 mg/dL, and serum potassium of 3.9 meq/L. The peak white blood cell (WBC) count was 8.38x10^3^/uL (reference: 4.5-13.5x10^3^/uL). The C-reactive protein (CRP) was <0.5 mg/dL (reference range: < 1 mg/dL). The dose of cefepime initiated was 2 g every eight hours. The blood culture subsequently resulted negative for infection. She continued to be observed and remained at her neurologic and psychologic baseline until the second day after cefepime administration, when the neurological symptoms began.

Diagnostic assessment

**Figure 1:**
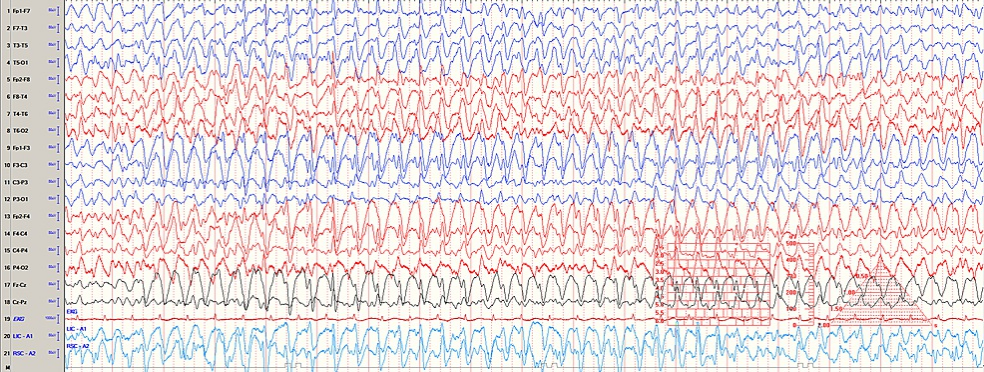
Abnormal EEG showing non-convulsive status epilepticus. EEG: electroencephalogram.

She was alert and followed commands appropriately. Her initial clinical manifestations included difficulty in walking and grasping items. This was soon followed by a short period of aphasia where she remained non-verbal and only responded to questions with head nods and shakes. Given her history of behavioral symptoms and depression, aphasia was challenging to assess. Physical exam findings were notable for sustained ankle clonus of the left lower extremity and intermittent rhythmic jerking movements of her extremities; however, these were easily ablated by touch and verbal distraction. She was reported having visual hallucinations on clinical exam, and cefepime was immediately discontinued as cefepime encephalopathy was the suspected diagnosis. Soon, she developed acute mental status change and had an episode of syncope while attempting to ambulate. Computed tomography (CT) of the head was performed to evaluate intracranial bleed or stroke, taking into account the risk of cefepime-induced INR (International Normalized Ratio) alteration with renal dysfunction. The CT scan did not show any acute abnormalities. Renal function labs were urgently obtained to assess possible electrolyte derangements contributing to the decompensation, which were notable for BUN of 48 mg/dL, serum creatinine 11.3 mg/dL, and serum potassium 5.7 meq/L. Given her end-stage kidney disease and previous pre-dialysis blood urea nitrogen baseline range of 30-50 mg/dL, uremic encephalopathy was considered low on the differential. Because of a normal WBC count, normal CRP, and negative blood culture, an infectious etiology was considered less likely. A continuous electroencephalogram (EEG) was placed to evaluate for the presence of seizures. EEG showed mild background slowing, multifocal and diffuse epileptiform discharges, generalized excessive beta intermittently, and slowing in the right posterior quadrant, left posterior quadrant, and central head regions (Figure 1). She was diagnosed with non-convulsive status epilepticus, believed to be related to excess accumulation of cefepime.

**Figure 2:**
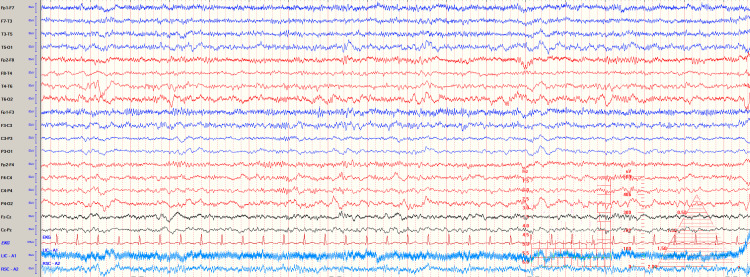
EEG showing resolution of status epilepticus following pharmacotherapy. EEG: electroencephalogram.

The epileptiform discharges on EEG were resolved following administration of lorazepam and levetiracetam load (Figure 2).

However, she still suffered from altered mental status and visual hallucinations despite AED therapy.

Therapeutic assessment

Upon review, a previous pharmacokinetic case-study in an adult patient with cefepime-induced encephalopathy showed an estimated cefepime clearance of 106.3 ml/min during hemodialysis and rapid resolution of encephalopathic symptoms following hemodialysis [6]. Urgent high-efficiency hemodialysis was ordered for our patient to treat cefepime-induced neurotoxicity given previous reports. She was started on hemodialysis using a high-efficiency dialyzer within three hours of the onset of neurological symptoms. The hemodialysis was performed using OptiFlux F160NR Dialyzer (advanced polysulfone membrane, the surface area of 1.5 m2, KUF of 45 ml/hr/mm Hg, and KoA urea of 1064). Throughout the course of the four-hour dialysis treatment, hallucinations and encephalopathic symptoms rapidly resolved. She received an additional Levetiracetam dose and 5 milligram/kilogram phenobarbital load for additional seizure activity noted on EEG after dialysis. By the following morning, and within 24 hours of initial presentation of neurological symptoms, she was back to her neurologic baseline with normal GCS (Glasgow coma score) and ambulating without assistance. She had normal speech, motor strength, reflexes, fine motor skills, and absence of clonus on clinical exam. She received three consecutive days of hemodialysis treatment before returning to her routine hemodialysis schedule three times weekly.

Additionally, a brain MRI was obtained the day after neurologic decompensation to evaluate for signs of posterior reversible encephalopathy syndrome (PRES), given her hypertension history, and it was unremarkable. The patient was continued on maintenance Levetiracetam throughout admission. She was discharged with instructions to continue maintenance Levetiracetam, 500 mg BID, and had outpatient neurology follow-up. There were no further reported seizures, and the patient continues to have normal neurological status after three months of close follow-up.

## Discussion

This case report, discussing a pediatric patient suffering from cefepime-induced encephalopathy and non-convulsive status epilepticus, highlights an under-reported population that can be affected by these adverse reactions. Cefepime causes cerebrospinal fluid (CSF) concentration-dependent GABA (γ-aminobutyric acid) antagonism in the central nervous system (CNS), leading to neurotoxicity [2]. Renal dysfunction is the primary risk factor of cefepime-induced encephalopathy [2,3]. In the setting of renal failure, there is impaired and delayed clearance of cefepime that increases the CSF concentration of cefepime [2,3,11]. Along with proteinuria and hypoalbuminemia that are seen in patients with renal failure, the protein binding of cefepime is decreased, which increases the passage of unbound cefepime across the blood-brain barrier [2]. The accumulation of organic acids in CSF in patients with renal failure inhibits the transport of cefepime from CSF to blood, further increasing the concentration of cefepime in CSF [2,8]. With normal kidney function, approximately 10% of serum cefepime crosses the blood-brain barrier, but this passage rises as high as 45% in the presence of renal dysfunction [2].

The common symptoms of cefepime neurotoxicity reported in adults include aphasia, myoclonus, visual hallucination, seizure, and non-convulsive status epilepticus [2-4]. In addition, in a pediatric patient, the presence of gait disturbance and ataxia was previously reported as a sign of cefepime neurotoxicity [8]. Similar to this previous report, our patient also had gait disturbance, which can be an early sign of cefepime neurotoxicity in pediatric patients. In addition, our patient also had aphasia and fine motor disturbance (difficulty grasping an object) as early symptomology. The diagnosis of cefepime neurotoxicity can be challenging based on early symptoms. Anti-epileptic therapy was effective in controlling the epileptiform discharges in the state of non-convulsive status epilepticus, as seen on continuous video EEG monitoring. This is a unique finding of our report. In this report, we highlight the importance of quickly ordering EEG monitoring to identify non-convulsive status epilepticus in patients with early signs of cefepime neurotoxicity.

In a pharmacokinetic study in children aged two months to 16 years with normal renal function, the mean half-life of cefepime was 1.7 hours (1.26-1.93 hours), Vd (volume of distribution) at a steady-state was 0.37 L/kg, low protein binding of 20%, renal total body clearance of 2.3 ml/min/kg, urine recovery of 72% and good CSF penetration [11]. Cefepime is excreted in the urine, primarily unchanged [12]. As renal function declines towards anuria or end-stage kidney disease, the half-life of cefepime increases from 2 hours to 13.5 hours [12]. Cefepime is a small molecule with a molecular weight of 480 Daltons, and it has pharmacokinetic properties of a low volume of distribution and poor protein binding that allow efficient removal by hemodialysis [6]. A high-efficiency dialyzer has a larger membrane surface area with KoA urea values of more than 800 and is more efficient to clear small molecules like cefepime. In one report, the use of high-efficiency hemodialysis generated a cefepime extraction fraction of 85.6%, and a cefepime clearance of 106.3 mL/min in an elderly patient with acute cefepime neurotoxicity, and the patient made a full neurological recovery 48 hours after hemodialysis [6]. Pharmacokinetic assessment in the same report showed a cefepime level returning to the non-toxic range 15 hours earlier with hemodialysis than without hemodialysis [6]. We performed urgent hemodialysis using a high-efficiency dialyzer (KoA urea 1064) to help accelerate neurological recovery after the diagnosis of cefepime neurotoxicity.

The primary treatment of cefepime neurotoxicity is the withdrawal of cefepime with symptomatic management. However, neurological recovery may be significantly delayed in the setting of renal dysfunction. In a previous report of cefepime neurotoxicity in a pediatric patient with acute kidney injury, the treatment consisted of removal of cefepime therapy, and complete neurological recovery occurred three days after discontinuation of cefepime [9]. In another report of cefepime neurotoxicity in a 14-year-old female with acute kidney injury, the serum cefepime level only decreased from 81 µg/mL to 62 µg/mL after more than four days, suggesting a significantly reduced clearance of cefepime with renal dysfunction. This patient remained in ICU for 17 days [10]. Our patient made complete neurological recovery within 24 hours of the initial presentation of neurological symptoms following high-efficiency hemodialysis.

In this case, our patient received an unadjusted renal dose of cefepime at the time of initial admission that likely contributed to cefepime encephalopathy along with the end-stage renal disease. The dosing adjustments in pediatric patients maintained on hemodialysis are suggested based on the pharmacokinetic studies in adult patients [13]. In a systematic review of 135 adult cases of cefepime neurotoxicity, 48% of patients received a dose of cefepime that was not adjusted for renal function. However, cefepime neurotoxicity also occurred in over 25% of patients with renal impairment who receive adjusted renal dosing of cefepime [2]. A retrospective study in adult patients with cefepime toxicity and end-stage renal disease failed to show a decrease in cefepime-induced encephalopathy with dose adjustments and encephalopathy was even seen with cefepime dose as low as 500 mg/day [3]. This highlights the importance of considering cefepime as a causative factor in acute mental decompensation when given in the setting of renal impairment.

One limitation of this case report is the lack of obtaining an objective cefepime level for diagnostic assessment as well as pharmacokinetic clearance study. However, given the elevated cefepime dosing regimen in our patient with end-stage renal disease as well as the rapid resolution of symptoms after high-efficiency hemodialysis and discontinuation of the offending agent, the diagnosis of cefepime-induced encephalopathy was established.

## Conclusions

Our purpose of this report was to discuss clinical findings of cefepime-induced neurotoxicity in a pediatric patient with renal dysfunction. While the early identification of cefepime neurotoxicity in pediatric patients is challenging, prompt EEG monitoring may help detect the presence of non-convulsive status epilepticus. Cefepime dosing should be adjusted for eGFR and dialysis status at the time of prescription writing. The primary treatment of cefepime-induced encephalopathy is the discontinuation of cefepime and symptomatic management, but high-efficiency hemodialysis may be considered in a pediatric patient with renal dysfunction and severe cefepime encephalopathy, as it may help accelerate neurologic recovery.
